# The Use of Prosthetic Stents in Tracheobronchial, Gastrointestinal, and Genitourinary Diseases

**DOI:** 10.1155/DTE.1.1

**Published:** 1994

**Authors:** Eric S. Edell, Rollin W. Hughes, Joseph E. Oesterling, Denis A. Cortese

**Affiliations:** 1 Division of Thoracic Diseases Mayo Clinic 200 First St. Southwest Rochester Minnesota 55905 USA; 2 Division of Thoracic Diseases Mayo Clinic Jacksonville FL USA; 3 Division of Gastroenterology Mayo Clinic Rochester MN USA; 4 Department of Urology Mayo Clinic Rochester MN USA

## Abstract

The concept of using a stent to maintain patency of a lumen is not new. As early as 1969, stents were being investigated in the peripheral arterial system as a means of preventing restenosis after dilatation by balloon angioplasty (Dotter, 1969). Since then, numerous reports have demonstrated the use of stents in both the peripheral and coronary artery systems (Maass et al., 1982; Dotter et al., 1983; Wright et al., 1985; Palmaz et al., 1987). Concomitant with the investigation of expandable endovascular metal prosthesis has been the development of prosthetic devices for management of tracheobronchial, gastrointestinal, and genitourinary diseases. We will review the use of endoscopically placed prosthetic devices in the management of diseases affecting these systems.


*Diagnostic and Therapeutic Endoscopy*, Vol. 1, pp. 1–8
Reprints available directly from the publisher
Photocopying permitted by license only

© 1994 Harwood Academic Publishers GmbH
Printed in Malaysia

### The Use of Prosthetic Stents in Tracheobronchial, Gastrointestinal, and Genitourinary Diseases

ERIC S. EDELL*, ROLLIN W. HUGHES, JR.†, JOSEPH E. OESTERLING†† and DENIS A. CORTESE**

**Division of Thoracic Diseases, Mayo Clinic, Rochester, MN*
***Division of Thoracic Diseases, Mayo Clinic, Jacksonville, FL*
*†Division of Gastroenterology, Mayo Clinic, Rochester, MN*
*††Department of Urology, Mayo Clinic, Rochester, MN*

*(Received in final form January 24, 1994)*

The concept of using a stent to maintain patency of a lumen is not new. As early as 1969, stents were being investigated in the peripheral arterial system as a means of preventing restenosis after dilatation by balloon angioplasty (Dotter, 1969). Since then, numerous reports have demonstrated the use of stents in both the peripheral and coronary artery systems (Maass et al., 1982; Dotter et al., 1983; Wright et al., 1985; Palmaz et al., 1987). Concomitant with the investigation of expandable endovascular metal prosthesis has been the development of prosthetic devices for management of tracheobronchial, gastrointestinal, and genitourinary diseases. We will review the use of endoscopically placed prosthetic devices in the management of diseases affecting these systems.

**KEY WORDS:** tracheobronchial stents, gastrointestinal stents, genitourinary stents, bronchial stricture, prostatic hypertrophy, biliary tract

### ENDOSCOPIC PLACEMENT OF PROSTHETIC DEVICES IN GENITOURINARY TRACT

Fabian (Fabian, 1980) was the first to describe the use of a stent or spiral in the lower urinary tract. He placed a metallic endoprosthesis in the prosthetic urethra of poor surgical risk patients in urinary retention as a result of an enlarged prostate. Based upon these results and the successful outcome of others, it became clear that *stenting* of the lower urinary tract was a workable concept (Fabricius *et al.*, 1983; Flier and Seppelt, 1987).

At the present time, there are a number of intraurethral endoprostheses being developed for use in the lower urinary tract. However, the two permanently implanted intraurethral stents that have been investigated most extensively in both Europe and the United States are the Intraprostatic Stent by advance surgical intervention (San Clemente, California), and the UroLume Endoprosthesis, which is being marketed by American Medical Systems (Minnetonka, Minnesota). The Intraprostatic Stent has been evaluated primarily as a treatment of bladder outlet obstruction secondary to an enlarged prostate gland. However, the UroLume Endoprosthesis has been investigated as a treatment option for three urologic conditions: 1) recurrent bulbar urethral strictures; 2) benign prostatic hypertrophy; and 3) detrusser external sphincter duissynergia. In the discussion that follows, the data relating to the role of the UroLume Endoprosthesis in the lower urinary tract are reviewed.

### Recurrent Bulbar Urethral Strictures

The initial experience with this permanently indwelling endoprosthesis in the treatment of recurrent bulbar urethral strictures comes from England. In 1988, Milroy and colleagues (Milroy *et al.*, 1988) reported their preliminary results on eight patients. All of these men (mean age: 57 years; range: 33–78 years) had been previously treated with a minimum of two direct vision internal urethrotomies (DVIU) and multiple urethral dilatations without success

Address for correspondence: Eric S. Edell, M.D., Division of Thoracic Diseases, Mayo Clinic, 200 First St. Southwest, Rochester, Minnesota 55905, U.S.A.

and were awaiting a formal urethroplasty. After first dilating the dense stricture to 30-French (1.0 cm), the UroLume Endoprosthesis was placed across the stenosed area.

In two patients (25%), the endoprosthesis was inserted under radiological control, and in six (75%), the stent was positioned in the bulbar urethra under direct vision using an optical telescope. With a mean followup of 8 months (range: 6 months–1 year), no patient developed a recurrent stricture. All men had total resolution of their obstructive symptoms and an excellent caliber urethra on followup cystoscopic examination. For the seven patients with urodynamic data available, the mean peak urinary flow rate increased from 8.3 ± 1.8 ml/second prestent placement to 22.6 ± 7.0 ml/second postprocedure ($p < 0.001$). The only side effect observed in this preliminary study was occasional post-micturation dribbling for several weeks after stent placement. In the following year, these same investigators presented a followup report on 12 patients (mean age: 59 years; range: 31–88 years) with recurrent bulbar urethral strictures who were treated with the UroLume Endoprosthesis (Milroy *et al.*, 1989). A total of 17 stents were inserted; 9 patients (75%) received one stent, one man (8%) had two stents, and 2 individuals (17%) had three stents inserted to completely cover the strictured area. With the followup period ranging from 2 to 13 months (mean: 7 months), no stricture recurred. For the 11 evaluable patients, the mean peak urinary flow rate increased by 200% (7.6 ± 2.3 ml/second pre-insertion to 22.8 ± 8.4 ml/second poststent placement). By 4 to 6 months after placement, the stents were completely covered with urothelium, and thus no longer exposed to the urine during micturation. Untoward effects included transient, mild discomfort at the stent site for 2 to 3 weeks in five men (42%); five other patients complained of mild postvoid dribbling after stent insertion. There was no evidence of encrustations or infection, and no patient complained of alteration in ejaculation or ability to achieve an erection.

Ashken and colleagues (Ashken *et al.*, 1991) reported the European experience with the UroLume Endoprosthesis as a treatment for difficult bulbar urethral strictures. With four urologic centers participating, 71 patients (mean age: 56 years; range: 30–81 years) were managed with the endoprosthesis over a 3-year period. The most common etiology was iatrogenic, and all patients had undergone multiple previous treatments without success. All men were followed for a minimum of 6 months, and 12 (17%) had been monitored for over 2 years after stent placement. Subjectively, 96% (68 patients) were pleased with the outcome. Objectively, the mean peak urinary flow rate increased from 6 ml/second pre-insertion to 20 ml/second in 3 months ($p < 0.01$), 18 ml/second at 8 months ($p < 0.01$), and 22 ml/second at 15 months ($p < 0.01$) post-stent placement. No patient developed a recurrent stricture. The authors, however, commented that none of the patients in the series had a traumatic stricture; they recommended that traumatic strictures, which can be associated with very dense fibrous tissue, should not be managed with the UroLume Endoprosthesis. As for the present series, the only notable untoward effects were urethral and perineal discomfort and occasional postvoid dribbling in the immediate postoperative period. There was no difficulty with infection or encrustations. The North American UroLume Study Group also evaluated the UroLume Endoprosthesis in a multicenter clinical trial (Oesterling *et al.*, 1993). One hundred and seventy-five patients (mean age: 52 years; range: 17–90 years) with recurrent bulbar urethral strictures underwent treatment with the UroLume Endoprosthesis. The mean number of previous treatments (urethral dilatation or DVIU) per patient was 18. Stricture length for the entire cohort varied from 0.4 cm to 5.5 cm (mean: 2.2 cm). The results at 12 months followup were most encouraging. The total symptom score decreased from 12.7 ± 5.1 pre-insertion to 2.1 ± 2.3 ($p < 0.001$). With voided volumes in excess of 150 cc, the peak urinary flow rate increased from 9.7 ± 6.1 cc/second pre-insertion to 22.2 ± 10.8 cc/second ($p < 0.001$). At 6 months post-insertion, 74% of the endoprostheses were completely covered with epithelium (Fig. 4). As of this time, there has been no difficulty with infection, erosion, encrustations, migration, continence, or potency. Several of the younger patients, however, have noted perineal discomfort with nocturnal erections in the postoperative period. For all such patients, this untoward effect resolved within 6 to 8 weeks. Approximately 50% of the patients did have a mild degree of postvoid dribbling. Twenty-six men (15%) have required a second treatment to manage the urethral stricture; sixteen patients underwent placement of a second endoprosthesis, eight patients had an endourethral resection, one patient was managed with urethral dilatation, and one person had laser ablation of the tissue regrowth. Six patients (3%) have undergone stent removal; all prostheses were removed without difficulty and subsequent sequelae. These preliminary results from both Europe and the United States suggest that the flexible, self-expanding intraurethral stent may be an effective treatment for recurrent bulbar urethral strictures. Investigations with more patients and extended followup (years), however, will be necessary to determine the true durability of the endoprosthesis and assess late complications.

### Benign Prostatic Hyperplasia

As for bulbar urethral strictures, the early experience with the UroLume Endoprosthesis in the treatment of BPH comes from England, where this device has been utilized since January 1989 in patients who are a poor surgical risk. In a series of 12 patients described by Chapple and associates, 11 (95%) were "fully satisfied" and able to urinate without difficulty during a mean followup of 8.2 months (range: 1–11 months) (Chapple *et al.*, 1990). The one dissatisfied patient complained of significant frequency and urgency; this phenomenon was found on subsequent urodynamic evaluation to be associated with severe, persistent detrusor instability. The mean peak urinary flow rate at followup for the 12 patients, 9 of whom were in urinary retention prior to stent placement, was 13.6 ± 4.7 ml/second. The postvoid residual urine volume after stent placement was minimal. With respect to untoward effects, all 12 patients experienced some degree of irritative voidings symptoms in the immediate postoperative period. These symptoms subsided markedly within 4 to 6 weeks.

In a more recent report of 45 surgically unfit patients treated with the UroLume Endoprosthesis, Milroy and colleagues, indicated that 42 (93%) were pleased with the outcome; all were "passing urine normally with sufficiently reduced residual urine volumes" (Milroy, 1991). Most patients, however, suffered urgency, frequency, and occasional urge incontinence following stent placement; these symptoms tended to resolve within several months, as the endoprosthesis became covered with epithelium. Most often, the stent becomes covered with urothelium within 4 to 6 months' time, which is somewhat faster than that which occurs in the bulbar urethra. At 6 to 9 months after stent placement, these investigators did notice a significant amount of hyperplastic tissue growing through the intertices of the wall of the stent. However, by 12 to 18 months post-stent insertion, this had subsided markedly and was of no concern. No encrustations were observed on any part of the endoprosthesis located within the prostatic urethra. However, if the proximal end of the stent is allowed to extend across the bladder neck into the bladder, "fine encrustations" can develop on this aspect of the device 6 to 12 months after placement. In the Milroy series, it has been necessary to remove five stents. All devices were removed without difficulty. Two were not positioned properly at the time of deployment and were removed immediately, and a second stent was inserted; two others were removed 4 weeks after placement, and one was taken out 11 months after insertion. The technique for removing an endoprosthesis after it has become covered with epithelium is as follows. Once the overlying epithelium has been resected with a low-current resectoscope, the stent is jarred from it's bed in the prostatic urethra with a forceps. The stent is then grasped approximately 0.5 cm from its distal edge and pulled gently; it will lengthen and decrease in diameter (much like a Chinese finger toy) so that it can be pulled inside a resectoscope sheet and removed without trauma to the urethra or the external urinary spinchter (Chapple and Milroy, 1989). McLoughlin and co-workers (McLoughlin *et al.*, 1990) described 19 major-risk patients, all of whom were in urinary retention, who underwent placement of the endoprosthesis in the prostatic urethra with local anesthesia only. All patients tolerated the procedure with minimal to no discomfort, and all were able to "void spontaneously" during the followup period (mean: 4 months; range: 3–7 months). Fifteen patients (79%) had severe urgency, frequency, or discomfort with urination for 3 to 4 days after stent placement. For most patients, this untoward effect resolved within 8 weeks. All stents were noted to be covered with epithelium after 4 months. At the 1993 annual meeting of the American Urological Association, Oesterling (Oesterling 1993) reported the results of the North American Clinical Trial. Ninty-five *healthy* patients (mean age: 68 ± 7.5 years) with obstructive BPH elected to be treated with the UroLume Endoprosthesis. Unlike most patients being considered for treatment with the device in Europe, these men were sexually active and could have readily undergone a transurethral resection of the prostate gland. All patients were evaluated prior to stent placement and in followup with a standard symptom questionnaire, peak urinary flow rate, postvoid residual urine volume, and cystoscopic examination. The patients found to have a large, median lobe or considered to have a hypotonic bladder were excluded from participation. The results at 12 months followup were excellent. The total symptom scored decreased from 15.0 ± 5.5 pre-insertion to 6.3 ± 5.8 ($p < 0.001$). With voided volumes in excess of 150 cc, the peak urinary flow rate increased from 8.6 ± 3.5 cc/second pre-insertion to 15.6 ± 6.2 cc/second ($p < 0.001$). The postvoid residual urine volume decreased from a 129 ± 14.6 cc to 24 ± 44 cc ($p < 0.001$). By 6 months, 55% of the endoprostheses were completely covered with epithelium; 45% still had some aspect of the stent exposed at the bladder neck. There was no significant difficulty with infection, erosion, migration, continence, or potency; 67% of the patients, however, had some irritative symptoms (urgency, frequency, or dysuria) for at least 1 month after stent placement. Nine stents have been removed; all prostheses were removed without injury to the external urinary sphincter or the urethra. These results from both Europe and the United States are encouraging. Nevertheless, additional studies with extended followup will be necessary to establish the true, long-term usefulness of this endoprosthesis in the management of symptomatic BPH. A prospective, randomized clinical trial, comparing the UroLume Endoprosthesis with transurethral section of the prostate gland, is now underway in the United States and Canada.

### Detrusor External Sphincter Dyssynergia

Supraspinal cord injuries most often result in some degree of detrusor external sphincter dyssynergia (Barkin *et al.*, 1983). The majority of patients develop significant detrusor-external sphincter dyssynergia and require theraputic intervention. The two standard treatments are: 1) anticholinergic therapy (to decrease uninhibited bladder contractions and improve vesical compliance) with clean intermittent catheterization, and 2) external striated sphincterotomy to reduce bladder outlet resistance. The former option is only a consideration if the patient has sufficient manual dexterity. The latter treatment, on the other hand, does not always result in a favorable outcome. The results of endoscopic external sphincterotomy have been variable; overall, the success rate of this procedure is approximately 79 to 90%, as destruction of the entire external urinary sphincter is not always technically easy (Whitmore *et al.*, 1978). Side effects, such as rectal dysfunction and hemorrhage, also can be significant. Thus, there is much interest in developing a more simple, yet reliable, approach to achieving a functional external sphincterotomy. To this end, the UroLume Endoprosthesis is being investigated both abroad and in the United States as an alternative treatment for detrusor-external sphincter dyssynergia. In 1990, Shaw and colleagues (Shaw *et al.*, 1990) described nine patients with complete quadraplegia and detrusor-external sphincter dyssynergia who were managed with the UroLume Endoprosthesis instead of sphincterotomy; followup ranged from 12 weeks to 10 months. In six men (67%), a 3-cm endoprosthesis was sufficient to completely traverse the external sphincter; three patients (33%) required additional stents. Eight patients (89%) had marked improvement in bladder emptying; the mean postvoid residual urine volume decreased from 268 ml prestent insertion to 92 ml following the procedure. The other patient did not achieve complete bladder emptying and is awaiting urinary diversion. No complications were encountered in this series. McInerney and associates (McInerney *et al.*, 1991) placed this endoprosthesis in 22 patients with neurological conditions resulting in significant detrusor-external sphincter dyssynergia. Fourteen (64%) of these men had undergone previous surgery on the outflow tract; eleven had had repeated unsuccessful external sphincterotomies, and three had undergone placement of an artificial urinary sphincter at the bladder neck. With a maximum follow-up of 1 year, 15 (68%) achieved complete voiding after the procedure; 3 (14%) developed bladder neck obstruction after stenting the external urinary sphincter. The endoprosthesis was of no value in the three patients who had an artificial urinary sphincter placed previously at the bladder neck. Chancellor (Chancellor *et al.*, 1993) most recently reported the results of the North American Clinical Trial evaluating the UroLume Endoprosthesis as a treatment for detrusor-external sphincter dyssynergia (Fig. 6A and 6B). One hundred and nineteen spinal cord-injured men with this condition, as verified by urodynamic evaluation, were managed with an endoprosthesis and followed for 3 to 21 months. Thirty-two patients (27%) required two prostheses to completely bridge the external urinary sphincter. Voiding pressures decreased from 77.7 ± 26.2 cm of water before prosthesis placement, to 40.7 ± 16.5 cm of water at 3 months, 30.2 ± 15.2 cm of water at six months, and 26.1 ± 13.4 cm of water at twelve months ($p = 0.001$). The postvoid residual urine volume decreased from 188.9 ± 128.9 ml preoperatively to 112.5 ± 128.6 ml at 12-months followup ($p = 0.008$). Bladder capacity, however, remained constant ($p = 0.3$). All the patients with a prosthesis in proper position were able to achieve spontaneous reflex voiding without constant dribbling. Encrustations, infection, erosion, pain, and obstructive hyperplastic epithelial overgrowth did not occur in any patient. No delirious effects were seen with regard to renal or erectile function. In five patients, however, it was necessary to remove the device because of stent migration. Based on these preliminary results, it appears that the flexible, epithelizing UroLume Endoprosthesis may be an effective treatment for select patients requiring an external sphincterotomy. Nevertheless, it is only through long-term follow-up of many patients that the real effectiveness of this device will be demonstrated. Ideally, a prospective, randomized clinical trial comparing the UroLume Endoprosthesis with the goal of standard care, the endoscopic external sphincterotomy, should be conducted.

### ENDOSCOPIC PLACEMENT OF PROSTHETIC DEVICES IN GASTROENTEROLOGY

Endoscopic placement of prosthetic devices has been shown to be effective in three areas of gastroenterology. This includes obstruction from esophageal carcinoma, malignant biliary tract obstruction, and obstruction of the pancreatic ducts.

### Esophageal Stenting

Adenocarcinoma of the esophagus continues to rise dramatically as a leading cause of cancer death worldwide. The presentation of a patient with esophageal cancer is frequently by symptoms due to obstruction. In planning therapy, the level of tumor involvement is important. The esophagus is divided into three segments. The upper, which extends from the cricopharyngeus to 23 cm from the dentures, the middle level, which extends from 24 cm to 32 cm, and the lower esophageal segment, which is from 33 cm to the junction of the stomach. Tumors of the upper and middle third are generally beyond surgical cure at the time of their clinical presentation. It is has been estimated that 15 to 20% of patients with advanced esophageal carcinoma should be considered for esophageal stenting. This is particularly so after surgery, radiotherapy, chemotherapy, and laser therapy have been utilized. The tubes that have been used are constructed of polyvinyl plastic, latex rubber, or silicone rubber. They may be reinforced by a metal spiral, and have a proximal funnel construction which facilitates endoscopic placement.

A self-expanding silicone-covered tube (Gianturco stent) was used for palliation of esophago-respiratory fistula related to esophageal cancer in eight patients by Do (Do *et al.*, 1993) at the Korea Cancer Center Hospital. All fistula were successfully occluded by these expanding tubes. The expanding tubes imbed themselves into the esophageal wall, and a significant tissue hyperplasia occurs, particularly at the edge of the tube.

Radiologists at the University Hospital, Lund, Sweden have implanted nitinol self-expanding stents in 40 patients with dysphagia (Cwikiel *et al.*, 1993). There were no immediate complications, although ingrowth of the tumor was seen in eight patients. Two of the patients had occlusion of the stent by tumor and required laser resection to re-establish luminal patency.

The Gianturco metallic stent was used by Iwasaki and collegues (Iwasaki *et al.*, 1993) to bridge a malignant stricture of a esophagojejunostomy. Truong and collegues (Truong *et al.*, 1992) also reported palliative treatment of malignant gastric outlet obstruction using a self-expanding metal stent.

**Biliary Duct and Pancreatic Duct Stenting**

The placement of biliary and pancreatic endoprostheses is also receiving intensive study and experimentation. Endoscopic stent placement has become accepted palliation for patients with inoperable malignant biliary tract obstruction. Specific conditions that may be amenable to stenting include pancreatic and biliary carcinoma, or metastatic lesions that either compress or infiltrate the biliary tree such as carcinoma of the gallbladder, carcinoma of the stomach, or cancers arising in the colon, breast, etc. The major condition that has benefitted from endoscopic stenting has been pancreatic adenocarcinoma. Of this group of patients, 22 percent also had malignant hilar obstruction.

The success rate for endoscopic placement of biliary stents is approximately 80 to 93% and the overall success of insertion is judged by evaluating complications encountered in obtaining the goals of the procedure. A preliminary goal in the treatment of malignant obstruction is the relief of jaundice. Kodakin and Starnes (Kodakin and Starnes, 1992) have compared the 10 French and the 11.5 French polyethylene biliary stents, and their effectiveness in obtaining relief of icterus, and decline of total bilirubin. They concluded that there is no significant advantage between these stent sizes. However, the stent patency is approximately twice as long for the 10 French polyethylene stent compared to the 7 French stent. There is a slight tendency for a higher complication rate using the larger stent (Pedersen, 1993).

The occlusion rate most certainly impacts on the acceptance of stenting of the biliary ductal system as palliative therapy. The occlusion rate for a 10 French or 11.5 French stent at 3 months is 42%, and at 6 months is 10.8% (Davids *et al.*, 1992). Furthermore, a worse clinical response and poorer survival is seen in the group of patients who have undergone stenting for metastatic cancer (Frakes *et al.*, 1993).

The search for a better stent is primarily based on the ease of insertion and stent patency. This search has led to the use of metal expandable stents. The University of Amsterdam investigators have compared an expandable metal stent with a polyethylene stent (Davids *et al.*, 1992). The median patency for the first stent insertion was significantly prolonged in patients with a metal stent compared with the polyethylene stent, 273 days versus 126 days, respectively. The major cause of stent dysfunction was tumor ingrowth in the metal stent group, and sludge deposit in the polyethylene group. When stent blockage was encountered, a polyethylene stent was inserted. Of those patients in the metal stent group, 14 required a polyethylene stent insertion, and no further occlusions were encountered. In the group who had a polyethylene stent initially inserted, 23 required an additional stent inserted. Eleven of the 23 (48%) who had a new polyethylene stent inserted had an additional episode of occlusion. This fact suggests that re-occlusion is more likely to occur when the endoscopist uses only polyethylene stents.

Since plastic stents are removable, they have been employed in the treatment of benign biliary strictures. A nonrandomized study by Davids (Davids *et al.*, 1993 retrospectively compared surgical management and endoscopic management of benign biliary strictures. Endoscopically placed biliary stents, which were exchanged every third month for a 1 year period, were compared to patients who were surgically managed. Seventeen percent had recurrent stricture after undergoing endoscopic dilatation and stenting. Another way that this result can be stated is that 83% of their strictures were patent after being submitted to endoscopic care, while the literature frequently states that surgical management of strictures has an 80% long-term success. The authors conclude that this practice scheme is an acceptable way to initially manage strictures, and that the surgical repair should be reserved for patients who have complete transection of the duct, failed previous surgical repair, or failed an endoscopic treatment program. This recommended approach seems to be an oversimplification of a very complex situation.

Millis (Millis *et al.*, 1992) retrospectively analyzed 194 patients who were consecutively treated at UCLA for bile duct strictures. Those seen from 1955 to 1979 were separately analyzed in group 1, and those seen from 1980 to 1990 were reviewed in group 2. The recurrent stricture rate was 21 and 23% respectively. The re-operation rate for group 1 was 21% and for group two, 6%. An explanation as to why there was a striking reduction in re-operation, was that 20 patients in group 2 underwent biliary dilatation. Yet, the success of biliary dilatation varies greatly depending upon the pathologic condition giving rise to stricturing. Ninety-three percent of those treated with anastomatic strictures were adequately cared for by dilatation, while only 50% of patients with a primary stricture benefited from dilatation.

Biliary endoprostheses have been used in elderly symptomatic patients with large bile duct stones. The use of a stent has proven to be an effective treatment for nonextractable stones. An excellent article by Chung (Chung *et al.*, 1991) from the Prince of Wales Hospital, Hong Kong, reviewed their experience clearing biliary ducts of large stones. Mechanical lithotripsy was successful in 81% and the remaining patients had their ducts cleared of calculi by electrohydraulic lithotripsy, surgery, or stenting.

The pancreas and its afflictions are being approached with the insertion of straight stents. Therapeutic endoscopists hope that bypassing obstructive lesions will diminish pain and restore or preserve function. Segmental stenotic lesions in the head region and intraductal calculi, that are not removable, are being stented. Polyethylene pancreatic duct stents induce changes in the pancreas of normal dogs (Sherman *et al.*, 1993). Similar changes have been observed in humans (Kozarek, 1990). It appears the cause of this damage is related to stent occlusion or from local ductal trauma. Due to this known complication, caution must be exercised in application of pancreatic stents.

### ENDOSCOPIC PLACEMENT OF TRACHEOBRONCHIAL PROSTHESES

Many patients with malignant airway obstruction present with disabling dyspnea that is not amenable to surgical intervention. Laser resection of tumor has provided palliation in some of these patients. Unfortunately, many patients have obstruction from extrinsic compression by tumor or enlarged lymph nodes. The development of endobronchial prosthetic devices may offer some of these patients an alternative to disabling dyspnea.

The concept of an airway prosthetic device is not new, but unfortunately, materials and techniques for insertion have been previously unsatisfactory. Development of products that are easer to insert and more tolerable to the patient has led to an expanded use of prosthetic devices in the management of airway obstruction. Several types of tracheobronchial prostheses are currently available (Clark, 1980; Cooper *et al.*, 1981; Dumon, 1990; Insall and Morritt, 1991; Montgomery, 1965; Montgomery, 1968 Neville *et al.*, 1972; Orlowski, 1987; Paliero and Shepherd, 1974; Simonds *et al.*, 1985; Uchida *et al.*, 1988; Wallace *et al.*, 1986 Westaby *et al.*, 1982; Westaby and Shepard, 1983). Three basic types of prosthetic devices are currently available: 1) molded silicone prostheses, 2) expandable metal prostheses, or 3) combinations of metal and silicone.

Dumon (Dumon, 1990) reported results of a silicone stent with studs on its external surface to prevent migration. In this series, a total of 118 prostheses were inserted in 66 patients. Immediate relief of respiratory symptoms with significant quality of survival was achieved in all but two cases. The prosthesis was placed in the trachea in 59 cases, the left mainstem bronchus in 34 cases, and the right mainstem bronchus in 16 cases. Obstruction of the prosthesis by dried secretion was recorded in two patients. Granulomas were noted in 10 cases. The authors report migration of the prosthesis in 12 patients.

Becker (Becker, 1992) reported experience with expandable metal prostheses, mainly the Strecker stent and the Palmaz stent. These prostheses are made of tantalum or stainless steel compressed onto balloons and expanded after introduction by inflation with saline solution. The expandable metal stents become epithelialized in the ideal situation. Becker reports 36 implantations of the Strecker stent with complications occurring in the majority of these patients. Complications included granulomatous formation with obstruction and integration into the bronchial wall. As a result of this experience, the author does not recommend use of these prosthesis for airway obstruction.

Self-expanding metal prothesis have also been used in the management of malignant or benign airway obstruction. Several authors have reported their experience with these devices (Bohndorf *et al.*, Rauber *et al.*, 1992). Becker reported implanation of 77 Nitinol stents. The complications appeared less than with the Strecker stents. Rousseau reported an experience with self-expandable type prosthetic devices. He reported 74 stents in 62 patients

(Rousseau *et al.*, 1992). Thirty-nine Wallstents (trachea 14, bronchi 25), and 35 Gianturco. This series represented consecutive patients with contraindications for surgery or presented with noninflammatory lesions of the trachea or bronchus. Rousseau reported complications in 6 of 19 patients (31%) with the Gianturco self-expanding stent.

At our institution, we have had the opportunity to use Dumon silicone stents and a specially designed Stent Introducer System (Bryan Copr, Woburn, MA.). Forty stents have been inserted into thirty patients. The majority of these patients had malignant airway obstruction. Other causes of airway narrowing included benign stricture, Wegener's granulomatosis, and mediastinal fibrosis. One patient has had the prosthetic device in place for more than 48 months. The major complication has been obstruction by inspisated secretions. One patient experienced stent dislodgement and one patient had a stent removed due to intractable cough. Our experience supports the use of silicone prostheses in selected patients with airway obstruction due to malignant or benign processes.

The ideal prosthetic device for tracheobronchial obstruction has not yet been witnessed. Depending on the circumstances, the silicone prosthesis of the Dumon type may have advantages over the self-expanding metal stents. Clearly, for the benign stricture in patients who are not surgical candidates, the silicone prosthesis is the instrument of choice. As more development occurs, the metal self-expanding stents may find more appropriate applications. The combined metal silicone stents that are currently under development may also provide a new class of stents that will be more appropriate for patients with airway obstruction.

### Summary

Endoscopic placement of endoluminal prosthetic devices continues to expand. Significant palliation can be accomplished in patients with diseases that effect not only the vascular system, but also the genitourinary, gastrointestinal, and tracheobronchial tree. Symptoms of recurrent bulbar urethral strictures, benign prostatic hypertrophy, and detrusor external sphincter dyssynergia may be managed in some patients using these new devices. Obstruction of the esophagus by carcinoma or obstruction of the biliary or pancreatic ducts may also be palliated using new prosthetic devices. Malignancies and benign processes causing airway obstruction with resulting dyspnea may be managed by dilatation and placement of prosthetic devices in the tracheobronchial tree. Although devices are available for each of the above conditions, further development and investigation needs to be undertaken to improve both methods of application as well as analyze long-term results in these disorders.

### REFERENCES

Ashken M. H., Coulange, C., Milroy, E. J. G., Sarramon, J. P.: European experience with the urethral Wallstent for urethral strictures. Eur Urol 1991; 19:181–185.

Barkin M., Dolfin, D., Herschorn, S., Bharatwal, N., Comisarow, R.: The urologic care of the spinal cord injured patient. J Urol 1983; 129:335–339.

Becker H. D.: Treatment of Central Airway Stenosis by Bronchoscopic Stent Application. Presented at The 7th World Congress for Bronchology, Rochester Minnesota. September 28–October 2, 1992.

Bohndorf K., Kureja, G., Schlondorff, D., Vorwerk, R. W.: Implantation selbstexpandierender Endoprothesen (Wallstent) bei benignen Trachealobstruktionen, in J. Kollath und D. Liermann (Hrsg.) Stents II, Schnetstor—Verlag, Konstanz, 1992; S.234–242.

Chancellor M. B., Ackman, C. F. D., Appell, R. A., Binard, J., Boon, T. B., Roehrborn, C. G., Chetner, M. P., Chetner, M., Thorndyke, W. C., Defalco, A., Mayo, M., Gajewski, J., Green, B., Bennett, J., Foote, J., Juma, S., Linsenmeyer, T., MacMillan, R., Stone, A., Vasquez, A.: Multicenter trial of North America of UroLume urinary sphincter prosthesis. J Urol 1993; 149:358A.

Chapple C. R., Milroy, E. G. J.: Use of stents for treating obstruction of urinary outflow (letter). BMJ 1989; 299:795.

Chapple C. R., Milroy, E. J. G., Rickards, D.: Permanently implanted urethral stent or prostatic obstruction in the unfit patient: Preliminary report. Br J Urol 1990; 66:58–65.

Chung S. C., Leung, J. W., Leong, H. T., Li, A. K.: The chemical lithotripsy of large common duct stones using a basket. Brit J Surg 1991; 78:1488–50.

Clarke D. B.: Palliative intubation of the trachea and main bronchi. J Thorac Cardiovasc Surg 1980; 80:736–741.

Cooper J. D., Todd, T. R. J., Ilves, R., *et al.*: Use of the silicone tracheal T-tube for the management of complex tracheal injuries. J Thorac Cardiovasc Surg 1981; 82:559–568.

Cwikiel W., Stridbeck, H., Tranberg, K. G., *et al.*: Malignant esophageal strictures: treatment with a self-expanding nitinol stent. Radiol 1993; 187:661–665.

Davids P. H., Groen, A. K., Rauws, E. A., *et al.*: Randomized trial of self-expanding metal stents versus polyethylene stents for distal malignant biliary obstruction. Lancet 1992; 340:1488–92.

Davids P. H., Tanka, A. K., Rauws, E. A., *et al.*: Benign biliary strictures. Ann Surg 1993; 217:237–243.

Do Y. S., Song, H. Y., Lee, B. H., *et al.*: Esophagorespiratory fistula associated with esophageal cancer: treatment with a Gianturco stent tube. Radiol 1993; 187:673–677.

Dotte, C. T., Buschmann, R. W., McKinney, M. K., Rösch, J.: Transluminal expandable nitinol coil stent grafting: preliminary report. Radiol 1983; 147:259–260.

Dotter C. T.: Transluminally-placed coilspring enarterial tube grafts: long-term patency in canine popliteal artery. Invest Radiol 1969; 4:329–332.

Dumon J. F.: A dedicated tracheobronchial stent. Chest 1990; 97:328–332.

Fabian K. M.: Der intraprostatische "Partielle Katheter" (Urologische Spirale). Urolge [A] 1980; 19:236–238.

Fabricius P. G., Matz, M., Zepnick, H.: Die endourethralspirale—eine alternative zum dauerkatheter? Z Arztl Fortbild (Jena) 1983; 77:482–485.

Flier G., Seppelt, U.: Einfahrungen mit der urologischen spirale. Urolge [B] 1987; 27:304–307.

Frakes J. T., Johanson, J. T., Stake, J. J.: Optimal timing for stent replacement in malignant biliary tract obstruction. Gastrointest Endosc 1993; 39:164–167.

Insall R. L., Morritt, G. N.: Palliation of malignant tracheal strictures using silicone T-tubes. Thorax 1991; 46:168–171.

Iwasaki T., Hayashi, N., Kimoto, T., *et al.*: Application of a self-expanding metallic stent to a strictured esophagojejunostomy. Cardiovasc and Intervent Radiol 1993; 19:98–101.

Kadakia S. C., Starnes, E.: Comparison of 10-French gauge stent with 11.5 French gauge stent in patients with biliary tract disease. Gastrointestinal Endoscopy 1992; 38:454–459.

Kozarek R. A.: Pancreatic stents can induce ductal changes consistent with chronic pancreatitis. Gastrointest Endosc 1990; 36:93–95.

Maass D., Kropf, L., Egloff, L., Demierre, D., Turina, J., Senning, A.: Transluminal implantations of intravascular "Double Helix" spiral prostheses: technical and biological considerations. Proc Eur Soc Artif Organs 1982; 9:252–254.

McInerney P. D., Vanner, T. F., Harris, S. A. B., Stephenson, T. P.: Permanent urethral stents for detruso sphincter dyssynergia. Br J Urol 1991; 67:291–294.

McLoughlin J., Jager, R., Abel, P. D., El Din, A., Adam, A., Williams, G.: The use of prostatic stents in patients with urinary retention who are unfit for surgery. Br J Urol 1990; 66:66–70.

Millis, J. M., Tompkins, R. K., Zinner, M. J., *et al.*: Management of bile duct strictures, an evolving study. Arch Surg 1992; 127:1077–82.

Milroy E. J. G., Chapple, C. R., Cooper, J. E., Eldin, A., Wallsten, H., Seddon, A. M., Rowles, P. M.: A new treatment of urethral strictures. Lancet 1988; 1:1424–1427.

Milroy E. J. G., Chapple, C. R., Eldin, A., Wallsten, H.: A new stent for the treatment of urethral strictures: preliminary report. Br J Urol 1989; 63:392–396.

Milroy E.: Permanent prostate stents. J. Endourol 1991; 5:75–78.

Montgomery W. W.: T-tube stent. Arch Otolaryngol 1965; 83:71–75.

Montgomery W. W.: The surgical management of supraglottic and subglottic stenosis. Ann Otol Rhinol Laryngol 1968; 77:534–546.

Neville W. E., Hamouda, F., Anderson, J., *et al.*: Replacement of the intrathoracic trachea and both stem bronchi with a molded silastic prosthesis. J Thorac Cardiovasc Surg 1972; 63:569–576.

Oesterling J. E., Defalco, A., and the North American UroLume Study Group: The UroLume Endoprosthesis as a treatment for recurrent bulbar urethral strictures: long-term results from the North American Clinical Trial. J Urol 1993; 149:505A.

Oesterling J. E., Epstein, H., and the North American UroLume Study Group: The North American experience with the UroLume Endoprosthesis for symptomatic BPH: long-term results. J Urol 1993; 149:216A.

Orlowski T. M.: Pallative intubation of the tracheobronchial tree. J Thorac Cardiovasc Surg 1987; 94:343–348.

Paliero K. M., Shepherd, M. P.: Use of stainless steel wire coil prosthesis in treatment of anastomatic dihescence after cervical tracheal resection. J Thorac Cardiovasc Surg 1974; 67:932–935.

Palmaz J. C., Kopp, D. T., Hayashi, H., Schatz, R. A., Hunter, G., Tio, F. O., Garcia, O., Alvarado, R., Rees, C., Thomas, S. C.: Normal and stenotic renal arteries: experimental balloon-expandable intraluminal stenting. Radiol 1987; 164:705–708.

Pedersen F. M.: Endoscopic management of malignant biliary obstruction. Is stent size of 10-French gauge better than 7-French gauge? Scand J Gastroenterol 1993; 28:185–189.

Rauber K., Weimer, B., Syed Ali, S., Kollath, J., Jochims, H.: Endotracheale NiTi-Stents—Tierexperimentelle Studie in J. Kollath und D. Liermann (Hrsg.) Stents II, Schnetztor—Verlag, Konstanz, 1992; S.243–250.

Rousseau, H., Dahan, M., Bilbao, I., Joffre, F.: Use of Expandable Proshteses in the Tracheo-bronchial Tree. Presented at; Vascular Stents 1992: A Comprehensive Workshop. LaJolla, California. June 4–6, 1992.

Shaw P. J. R., Milroy, E. J. G., Timoney, A. G., Eldin, A., Mitchell, N.: Permanent external striated sphincter stents in patients with spinal injuries. Br J Urol 1990; 66:297–302.

Sherman S., Alvarez, C., Robert, M., *et al.*: Polyethylene pancreatic duct stent-induced changes in the normal dog pancreas. Gastrointest Endosc 1993; 39:658–664.

Simonds A. K., Irving, J. D., Clarke, S. W., *et al.*: Use of expandable metal stents in the treatment of tracheobronchial malignancy. Otolaryngol Head, Neck Surg 1985; 93:205–210.

Truong S., Bohndorf, V., Geiller, H., *et al.*: Self-expanding metal stents for palliation of malignant gastric outlet obstruction. Endoscopy 1992; 24:443–435.

Uchida B. T., Putnam, J. S., Rosch, J.: Modifications of Gianturco expandable wire stents. AJR 1988; 150:1185–1187.

Wallace M. J., Charnsangavej, C., Ogawa, K., *et al.*: Tracheobronchial tree: Expandable metallic stents used in experimental and clinical applications. Radiol 1986; 58:309–312.

Westaby S., Jackson, J. W., Pearson, F. G.: A bifurcated silicone rubber stent for relief of tracheobronchial obstruction. J Thorac Cardiovasc Surg 1982; 83:414–417.

Westaby S., Shepard, M. P.: Palliation of intrathoracic tracheal compression with silastic tracheobronchial stent. Thorax 1983; 38:314–315.

Whitmore W. F., Fam, B. A., Yalla, S. V.: Experience with anteromedian (12 o'clock) external urethral sphincterotomy in 100 male subjects with neuropathic bladders. J Urol 1978; 50:99–101.

Wright K. C., Wallace, S., Charnsangavej, C., Carrasco, C. H., Gianturco, C.: Percutaneous endovascular stents: an experimental evaluation Radiol 1985; 156:69–72.

